# Making Sense of the Urgent GP Referrals; Audit Into How Many Are Actually Urgent?

**DOI:** 10.1192/bjo.2022.450

**Published:** 2022-06-20

**Authors:** Minahil Junaid, Bassem Habeeb

**Affiliations:** 1 Betsi Cadwaladr University Health Board, Wrexham, United Kingdom; 2 Wrexham Maelor Hospital, Wrexham, United Kingdom

## Abstract

**Aims:**

The trust policy dictates that all urgent GP referrals should be contacted within 48 hours by the duty team. The duty team carries out a telephone screening assessment and offers the patients who are deemed to be urgent, a face-to-face assessment. Those who are not assessed to be urgent are signposted to the right service.

**Methods:**

All the urgent GP referrals of the month of July 2021 were followed up retrospectively and the outcome was recorded to assess the influx and outcome of urgent referrals from primary care. The urgent referrals from all other routes such as Psychiatric Liaison, and Social Services, Police etc were not included in the data.

**Results:**

A total of 124 urgent referrals were received in the month of July 2021. Only 13 out the 124 were deemed urgent following the telephone assessment and they were offered a face-to-face assessment. Fifty three patients were referred to primary care mental health team, 24 were referred to the secondary community mental health, 20 were referred to the older adults team and 10 were discharged back to the GP following. Out of the 13 who were assessed by the duty team, 6 patients were referred to primary care mental health team and 6 were referred to the secondary community mental health team. The urgent referrals came from 20 GP surgeries that cover a wide area of the rural and urban communities and the surgeries with most urgent referrals were highlighted

**Conclusion:**

Trying to work on improving the quality of urgent referrals, the team tried to analyse the results, which proved to be complicated. The efforts to standardise the referral process has depended mainly on the degree of awareness of the GPs about the way the mental health service operates considering there is a percentage of locum GPs who might not be fully aware of how mental health service works.

The recommendation of the audit is to arrange visits to the GP surgeries to work on raising awareness among GPs about the referral system to the Mental Health team. It is also recommended that the GPs should be able to complete a brief risk assessment to justify why the referred patient needs to be reviewed urgently instead of on routine basis.

